# Association between nonspecific interstitial pneumonia and presence of CD20+ B lymphocytes within pulmonary lymphoid follicles

**DOI:** 10.1038/s41598-017-17208-1

**Published:** 2017-12-05

**Authors:** Min Peng, Wenze Wang, Ling Qin, Hongrui Liu, Mingwei Qin, Wenjie Zheng, JuHong Shi, Wenbing Xu, Yuanjue Zhu

**Affiliations:** 1 Division of Respiratory Medicine, Peking Union Medical College Hospital, Chinese Academy of Medical Sciences & Peking Union Medical College, Beijing, China; 2 Department of Pathology, Peking Union Medical College Hospital, Chinese Academy of Medical Sciences & Peking Union Medical College, Beijing, China; 3 Department of Internal Medicine, Peking Union Medical College Hospital, Chinese Academy of Medical Sciences & Peking Union Medical College, Beijing, China; 4 Division of Radiology, Peking Union Medical College Hospital, Chinese Academy of Medical Sciences & Peking Union Medical College, Beijing, China; 5 Division of Rheumatology, Peking Union Medical College Hospital, Chinese Academy of Medical Sciences & Peking Union Medical College, Beijing, China

## Abstract

Nonspecific interstitial pneumonia (NSIP) is characterised by interstitial infiltration of lymphocytes and varying amounts of interstitial fibrosis. B cells have been suggested to contribute to the pathogenesis of NSIP. However, the relationship between B-lymphocyte and the clinical outcomes of NSIP was unclear. In this study, 50 patients with histopathologically confirmed NSIP from Peking Union Medical College Hospital between April 2003 to December 2012 were retrospectively analyzed. Using immunohistochemical analyses, CD20+ B cells were counted in the lymphoid follicles, perivascular, interstitial, and peribronchiolar regions of lung tissure. The CD20+ lymphocytes were mainly present in the lymphoid follicles. The number of follicular CD20+ lymphocytes was higher in the fibrosing than cellular NSIP pattern [255.08 (132.92–449.71) vs. 121.33 (63.54–282.88)/0.1 mm^2^, p = 0.017]. After 1 year of therapy, the follicular CD20+ lymphocytes were significantly higher in patients whose forced vital capacity (FVC) worsened as compared to those who improved (p = 0.014). Additionally, follicular CD20+ lymphocytes were negatively correlated with the post-treatment percentage change in FVC (rho = −0.397, p = 0.004). However, follicular CD20+ lymphocytes were not correlated with survival. These results suggested that pulmonary follicular CD20+ lymphocytes were correlated with the fibrosing pattern of NSIP and predicted less clinical improvement after treatment.

## Introduction

Nonspecific interstitial pneumonia (NSIP) is an interstitial lung disease (ILD) that may be idiopathic or secondary to connective tissue disease, toxins, or numerous other causes. Although its aetiology and clinical course are highly heterogeneous, NSIP is histopathologically characterised by lymphocytic interstitial infiltration with occasional foci of fibroblasts and variable collagen deposition^1^. The precise mechanism of NSIP is unclear, but inflammation appears to be consistently present in the lungs of affected patients^2^. Thus, medical treatments for NSIP are typically based on glucocorticoids with or without cytotoxic agents.

Besides their capacity for antibody secretion, B lymphocytes act as antigen-presenting cells for T lymphocytes, provide additional co-stimulatory signals, and produce diverse inflammatory and regulatory cytokines^3,4^. CD20 is expressed on B cells beginning in the late pre-B-cell stage in the bone marrow and maintained during B-cell differentiation and development in the periphery. The expression of surface CD20 is then down-regulated on antibody-secreting plasmablasts and extinguished on plasma cells^5^. B lymphocytes are associated with a number of inflammatory respiratory diseases such as asthma, chronic obstructive pulmonary disease (COPD), hypersensitivity pneumonitis, sarcoidosis, and lung transplant rejection^4,6^. B lymphocytes also play a role in the development of autoimmune disease, and B-cell-targeted therapies are effective in the treatment of human autoimmune diseases^5^.

Studies of B-cell involvement in ILD are more limited, but examinations of diseased lungs from these patients have shown the presence of highly abnormal intrapulmonary B-cell aggregates^6–12^. Animal model studies have indicated a potential link between B-cell hyperactivity and fibrosis^13^. Lymphocyte aggregates comprising B and T lymphocytes are present in patients with idiopathic pulmonary fibrosis (IPF)^7,8^, and active cellular inflammation continues in IPF even in its severe end stage^9^. Infiltration of B and T lymphocytes is also present in the lungs of patients with idiopathic NSIP^10,11^, rheumatoid arthritis-associated NSIP and usual interstitial pneumonia^6,12^. However, the precise mechanism of B-cell involvement in NSIP remains unknown. Additionally, no studies have evaluated the relationship between B-lymphocyte infiltration in lung tissue and the clinical outcomes or progression of NSIP.

In this study, we investigated the potential importance of B cells in NSIP. We hypothesised that B-cell infiltration in the lung may be correlated with the clinical course of NSIP. Using immunohistochemical analyses, CD20+ B cells were quantified in stored tissue specimens from patients with NSIP. The relationship between the B-cell distribution and clinical outcomes was explored.

## Patients and Methods

### Patients and Diagnostic Criteria

From April 2003 to December 2012, 97 patients from Peking Union Medical College Hospital were diagnosed with NSIP based on surgical lung biopsies and attended follow-up appointments for pulmonary function testing and chest computed tomography (CT) scans. Lung specimens from 55 patients were available for further immunohistochemical analyses, while the other 42 patients were referral cases from other hospitals. Of the 55 patients, 5 were excluded due to use of immunosuppressive therapies before lung biopsy. The remaining 50 patients were included in this study. Their clinical features, radiological images, and pathological findings were reviewed and analysed.

NSIP was diagnosed based on pathological findings according to the American Thoracic Society/European Respiratory Society consensus classification^14^. NSIP was classified into three clinical subtypes: connective tissue disease (CTD)-associated NSIP (CTD-NSIP), autoantibody-positive NSIP, and idiopathic NSIP. Patients with NSIP who met the American College of Rheumatology criteria for CTD were assigned to the CTD-NSIP group^15–20^. Patients with NSIP who exhibited autoimmune features and had autoantibody positivity but did not meet the American College of Rheumatology criteria for CTD were assigned to the autoantibody-positive NSIP group. Patients were considered to be autoantibody-positive if they had an antinuclear antibody titre of >1:320 as well as positive results for anti-Sjögren’s syndrome antigen A, anti-Sjögren’s syndrome antigen B, anti-Scl-70, anti-Sm, anti-Jo-1, anti-ribonucleoprotein antibody, anti-keratin antibody, anti-perinuclear factor, or anti-cyclic citrullinated peptide antibody.

### Clinical Characteristics

We extracted the following clinical characteristics, which were documented at the first visit: age, sex, symptoms at the time of surgical lung biopsy (i.e., cough, dyspnoea, or wheezing), smoking status, physical examination findings, pulmonary function test results, and serologic antibody test results.

### Pulmonary Physiological Assessments

Forced vital capacity (FVC) via spirometry, total lung capacity via plethysmography, and diffusing capacity of the lung for carbon monoxide (DLCO) were measured according to the American Thoracic Society recommendations^21–23^. The results are expressed as percentages of the normal predicted values. We classified the patients into the following groups according to changes in FVC (% predicted values) after 12 months of steroid therapy: the FVC improved group exhibited FVC improvements of >10%, the FVC stable group exhibited FVC changes of 10% to −10%, and the FVC worsened group exhibited FVC that worsened by <−10%^24^.

### High-Resolution CT Scanning

High-resolution CT (HRCT) scans of the chest were performed on all patients at the initial evaluation. The HRCT scans were reviewed by an experienced chest radiologist with expertise in diffuse parenchymal lung disease. The radiologist was blinded to the patients’ identities and outcomes.

The specific HRCT findings were documented for the index scan. The extent of ground-glass opacity (GGO), reticulation, consolidation, and honeycombing were scored on a scale of 5% for all lobes. Honeycombing was defined as clustered cystic air spaces of 3 to 10 mm in diameter with shared well-defined walls and layering in the subpleural areas of the lungs^25,26^. The treatment response was assessed according to the CT findings after 1 year of steroid therapy. Patients with NSIP were classified as follows: in CT group 1, CT exhibited >50% improvement in ground-glass and reticular opacities; in CT group 2, CT exhibited <50% improvement; and in CT group 3, CT exhibited stable or worse findings^25–27^.

### Lung Tissue Histology

The lung tissue specimens were independently reviewed by two senior pathologists, and consensuses were reached regarding the histologic patterns. Fifty cases were pathologically subclassified as cellular or fibrosing patterns^2^. Small airways were defined as those with internal diameters of <2 mm and without cartilage. Small blood vessels were defined as those with an internal diameter of <100 μm^28^. The lung biopsy specimens were fixed in 10% neutral-buffered formalin solution, cut into slices, embedded in paraffin, and sectioned at 4-μm thickness for histologic evaluation. The paraffin sections were immunohistochemically stained manually with anti-CD14 antibody (clone EP128; Zeta Corp., Sierra Madre, CA, dilution 1:100) and anti-CD20 antibody (clone L26; Zeta Corp., dilution 1:100). The sections were deparaffinised and rehydrated with Tris-buffered saline (0.005 M Tris, 0.15 M NaCl) at pH 7.6 for 10 min. Endogenous peroxidase was blocked with 3% hydrogen peroxide for 5 min. The sections were then washed in Tris-buffered saline and incubated with primary antibodies at the appropriate dilutions for 1 h, and then incubated with secondary antibody for 30 min, finally detected by DAB (CD20 stain the B-cells while CD14 stain the monocytes).

**Figure 1:**
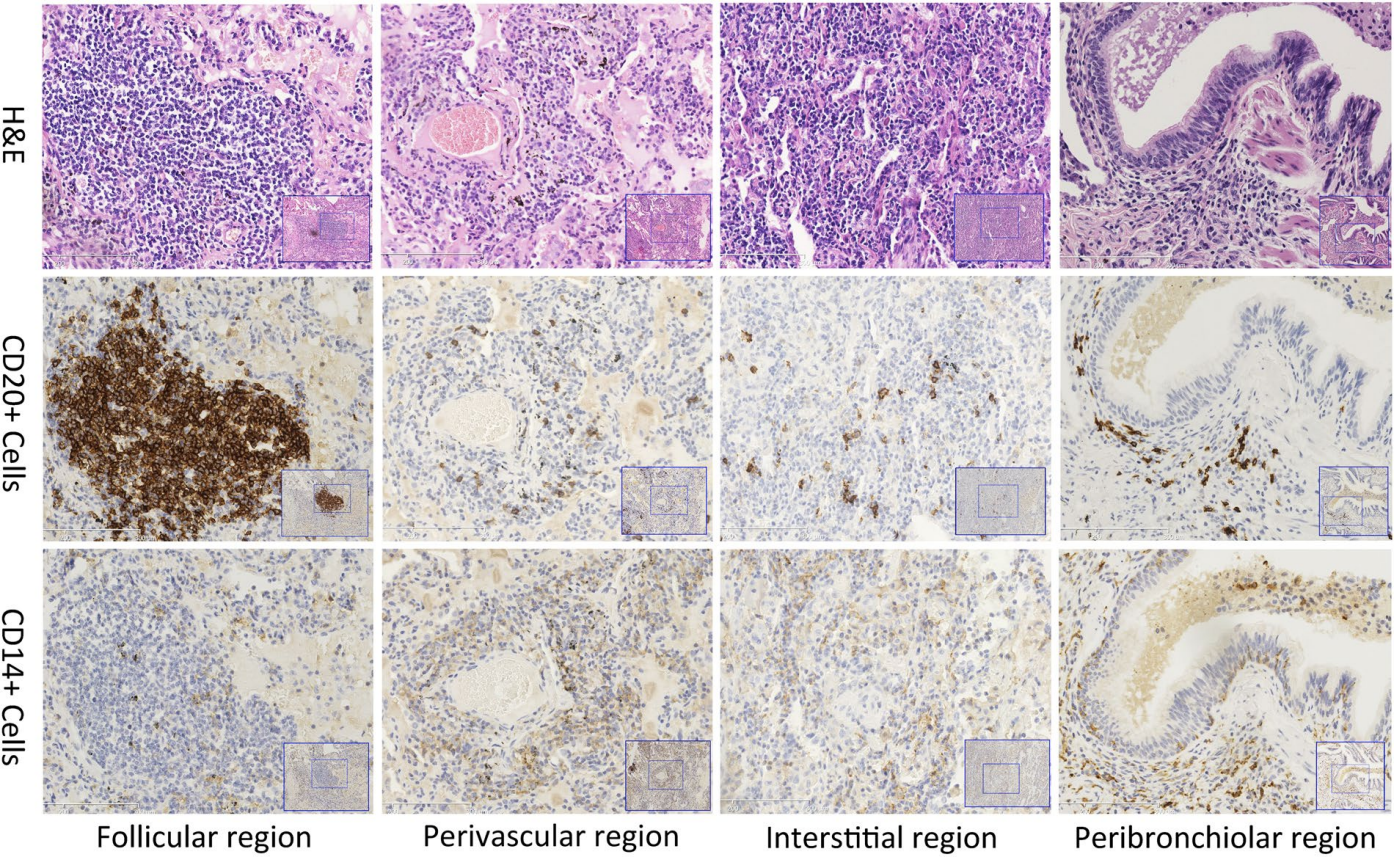
Distribution of lymphocytes in different anatomic regions of lung tissue. CD20+ and CD14+ lymphocytes mainly infiltrated the lymphoid follicle regions. H&E, haematoxylin and eosin.

The number of cells that were positively stained dark brown was analysed using the NanoZoomer 2.0-RS slide scanning system (Hamamatsu Photonics KK, Hamamatsu, Japan) and the Anymicro DSS Pro Image Analysis System (YuTianShiJiWeiYe Inc., Beijing, China). Four microscopic regions were analysed (i.e., the lymphoid follicles and the perivascular, interstitial, and peribronchiolar regions). Each biopsy slide contained all four of these microscopic regions. CD14 and CD20 images were obtained from the same region of the slide (Fig. 1). At least six high-power fields (magnification ×200, analysis area of approximately 0.162 mm^2^) were randomly selected for each region and used for cell counting.

### Treatment and Follow-up

After receiving a diagnosis of NSIP, all patients received a course of oral prednisone that began at 0.5 to 1.0 mg/kg/d for 1 month and was tapered every 3 weeks to 5.0 to 7.5 mg/d (the dose was decreased by 10% of the initial dose every 3 weeks); the dose was then maintained at 5.0 to 7.5 mg/d.

After the surgical lung biopsies, the patients underwent follow-up assessments every 3 months. These follow-ups included a complete blood cell count, liver function tests, and renal function tests. Chest CT scans and pulmonary function tests were performed at 3, 9, and 12 months after treatment at the Interstitial Lung Disease Clinic of Peking Union Medical College Hospital.

### Statistical Analyses

All values are expressed as mean ± standard deviation for normally distributed data and median (25–75%) for non-normally distributed data. The rank sum test was used for non-normally distributed data. The Wilcoxon signed rank test and the Kruskal–Wallis H test were used to compare two groups and more than two groups of data, respectively. Correlation coefficients were calculated using Spearman’s rank method. First, univariate analysis (the Kaplan–Meier method for categorical variables and a Cox proportional hazard regression model for continuous variables) was performed to explore the factors influencing survival, including age, sex, baseline FVC and DLCO, follicular CD20+ B lymphocytes, presence of CTD, presence of antibodies, clinical subtype of NSIP, and pathologic subtype of NSIP. Next, a Cox proportional hazard regression model (backward method, Wald test) was performed including the variables selected by the univariate analysis. Overall survival was calculated by Kaplan–Meier estimation. The Kaplan–Meier method and log-rank test were used to compare the survival of two groups. Probability values were obtained from two-sided tests, and statistical significance was set at p < 0.05. SPSS for Windows, version 23.0 (SPSS Inc., Chicago, IL, USA) was used for the statistical analyses.

Informed consent for use of the patients’ medical records was obtained from every patient and/or their guardian when the patient was admitted to the hospital. This study was approved by the Peking Union Medical College Hospital Institutional Review Board (reference number: 2013-9-322). All experiments were performed in accordance with relevant guidelines and regulations.

### Data Availability

The datasets generated and/or analysed during the current study are available from the corresponding author on reasonable request.

## Results

### Clinical Features and Laboratory Findings

Fifty patients with NSIP were included. Their clinical, radiologic, and physiologic measurements, which were obtained at the time of the initial visit, are shown in Table 1. Their mean age was 49.2 ± 10.7 years (range, 23–68 years), and 33 (66%) of the patients were women. The patients comprised 43 (86%) nonsmokers, 5 (10%) ex-smokers, and 2 (4%) current smokers. The median duration from the onset of respiratory symptoms to lung biopsy was 6 months (range, 1.0–36 months). Twenty patients were diagnosed with CTD-NSIP (polymyositis/dermatomyositis, n = 7; rheumatoid arthritis, n = 7; Sjögren’s syndrome, n = 4; and systemic sclerosis, n = 2). Twelve patients were serologically positive for at least one autoantibody but did not meet the diagnostic criteria for CTD, and 18 patients who were serologically negative were diagnosed with idiopathic NSIP.

**Table 1:** Demographic and clinical features of 50 patients with NSIP.

	NSIP (n = 50)
**Demographics**	
Age (years)	49.2 ± 10.7
Male, N (%)	17/50 (34)
Duration from disease onset to biopsy (months), median (range)	6 (1.0~36)
Follow-up time (months), median (range)	75.5 (8~147)
**Clinical manifestations, N (%)**	
Dry eyes or dry mouth	8/50(16%)
Fever	6/50 (12%)
Arthralgia	9/50 (18%)
Skin rash	6/50 (12%)
Raynaud’s phenomenon	3/50 (6%)
Weight loss	4/50 (8%)
Chest pain	4/50 (8%)
Cough	37/50 (74%)
Dyspnea	40/50 (80%)
Crackles	37/50 (74%)
Clubbing finger	17/50 (34%)
**Clinical subtype of NSIP, N (%)**	
CTD-NSIP	20
Antibody positive NSIP	12
Idiopathic NSIP	18
**Lab testing**	
ESR, mm/h	21.8 ± 20.0
PaO_2_, mmHg	77.7 ± 13.3
PCO_2_, mmHg	37.9 ± 8.4
**Baseline pulmonary function tests**	
FEV1.0, % predicted	68.6 ± 15.4
FVC, % predicted	67.1 ± 15.2
TLC, % predicted	76.8 ± 14.1
DLCO, % predicted	56.4 ± 16.8
**Baseline chest CT findings, N (%)**	
Ground glass opacity	33/50 (66)
Patchy opacity	35/50 (70)
Reticular opacity	32/50 (64)
Traction bronchiectasis	16/50 (32)
Pleural thickening	7/50 (14)

### Distribution of CD20+ Lymphocytes in Lung Compartments

Haematoxylin and eosin staining and CD20 staining of lung specimens revealed that infiltration of CD20+ lymphocytes was mainly concentrated in lymphoid follicle regions (Fig. 1). Table 2 shows that higher numbers of CD20+ lymphocytes infiltrated the lymphoid follicle regions than other anatomic regions [lymphoid follicles, 209.42 (94.46–347.79)/0.1 mm^2^; perivascular regions, 12.58 (5.92–24.08)/0.1 mm^2^; interstitial regions, 7.42 (3.29–15.50)/0.1 mm^2^; and peribronchiolar regions, 6.67 (2.63–11.08)/0.1 mm^2^; p = 0.000]. CD14+ lymphocytes were similarly distributed [41.75 (28.29–60.46)/0.1 mm^2^ in the follicles, 22.83 (16.13–33.88)/0.1 mm^2^ in the perivascular regions, 16.92 (12.71–29.54)/0.1 mm^2^ in the interstitial regions, and 14.42 (10.00–21.79)/0.1 mm^2^ in the peribronchiolar regions; p = 0.000)].

**Table 2:** Distribution of CD20+ and CD14+ lymphocytes in different anatomic regions.

Lymphocyte (N = 50)	Follicular region	Perivascular region	Interstitial region	Peribronchiolar region	*p* value
CD20+ cells (counts/0.1mm^2^)	209.42 (94.46–347.79)	12.58 (5.92–24.08)*	7.42 (3.29–15.50)**	6.67 (2.63–11.08) ***	0.000
CD14 + cells (counts/0.1mm^2^)	41.75 (28.29–60.46)	22.83 (16.13–33.88)*	16.92 (12.71–29.54)**	14.42 (10.00–21.79)***	0.000

*Follicular region vs. perivascular region, p = 0.000. **Follicular region vs. interstitial region, p = 0.000; ***Follicular region vs. peribronchiolar region, p = 0.000.

### Distributions of CD20+ and CD14+ Lymphocytes in Pathologic Subtypes of NSIP

According to the histopathologic subset criteria for NSIP, 20 patients with cellular NSIP and 30 with fibrosing NSIP were analysed. The features of the distributions of CD20+ and CD14+ lymphocytes in different histopathologic patterns of NSIP are shown in Table 3. The CD20+ lymphocyte infiltration in the lymphoid follicles of the patients with cellular NSIP [121.33 (63.54–282.88)/0.1 mm^2^] was less dense than in patients with fibrosing NSIP [255.08 (132.92–449.71)/0.1 mm^2^, p = 0.017]. No significant difference in CD14+ cell infiltration in the lung tissue was observed between cellular and fibrosing NSIP.

**Table 3:** Distribution of CD20+ and CD14+ lymphocytes in different pathologic subtypes of NSIP.

Lymphocytes (counts/0.1 mm^2^)	Pathologic subtype of NSIP		*p* value
	Cellular (n = 20)	Fibrosing (n = 30)	
**CD20+ Lymphocytes**			
Follicular region	121.33 (63.54–282.88)	255.08 (132.92–449.71)	**0.017**
Perivascular region	15.42 (7.04–36.83)	8.25 (5.08–20.88)	0.181
Peribronchiolar region	6.75 (3.04–9.33)	6.67 (1.42–12.33)	1.000
Interstitial region	9.08 (2.46–22.96)	6.32 (3.29–12.80)	0.367
**CD14 + Lymphocytes**			
Follicular region	41.67 (22.50–68.04)	42.25 (32.33–60.46)	0.649
Perivascular region	23.08 (20.00–35.38)	22.17 (15.50–31.33)	0.513
Peribronchiolar region	13.17 (8.88–20.58)	15.83 (10.46–22.25)	0.476
Interstitial region	18.92 (14.13–29.21)	15.65 (9.04–29.96)	0.470

### CD20+ Lymphocytes in Lung Tissue and Pulmonary Function Test Results

**Figure 2:**
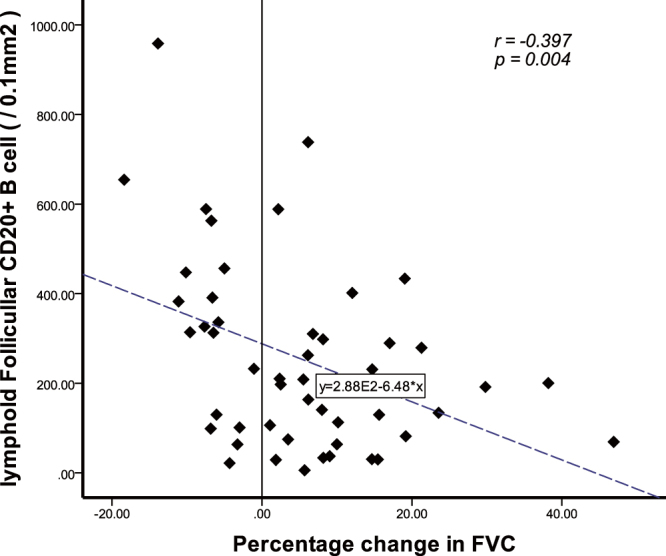
Negative relationship between follicular CD20+ B cells and percentage change in forced vital capacity after 12 months of therapy. FVC, forced vital capacity.

After 1 year of therapy, 19 of the 50 patients exhibited FVC improvement (% predicted values) of >10% (FVC improved group), 23 patients exhibited FVC changes (% predicted values) of 10% to −10% (FVC stable group), and 8 patients exhibited FVC changes (% predicted values) of <−10% (FVC worsened group). The follicular CD20+ lymphocyte counts showed a trend of gradual elevation from the FVC improved group to the FVC stable group and finally to the FVC worsened group (Table 4). The follicular CD20+ lymphocyte counts in the FVC worsened group were significantly higher than those in the FVC improved group [354.50 (175.83–637.96) vs. 140.67 (69.20–279.17), respectively; p = 0.014] (Table 4). In addition, the percentage change of FVC before and after treatment was negatively correlated with the follicular CD20+ lymphocyte count (rho = −0.397, p = 0.004) (Fig. 2). The densities of both the CD20+ and CD14 + lymphocytes that had infiltrated other anatomic compartments were not associated with the pulmonary function test results.

**Table 4:** Relationship between CD20+ lymphocytes and changes in FVC after 1 year of treatment.

CD20+ Lymphocytes (counts/0.1 mm^2^)	Changes of FVC^§^			*p* value
	FVC improved Group (n = 19)	FVC stable Group (n = 23)	FVC worsened Group (n = 8)	
Follicular region	140.67 (69.20–279.17)^*^	210.33 (74.67–391.17)^**^	354.50 (175.83–637.96)	**0.050**
Perivascular region	12.67 (6.17–36.00)	8.33 (2.50–21.50)	18.50 (9.89–46.54)	0.124
Interstitial region	8.50 (3.33–13.67)	7.17 (1.50–15.33)	8.75 (5.70–18.89)	0.607
Peribronchiolar region	6.50 (4.67–7.83)	6.67 (1.67–13.00)	7.42 (4.21–12.58)	0.958

^§^Changes in FVC (%predicted values) after 12 months of therapy. FVC improved group, FVC improvement of >10%; FVC stable group, FVC changes of 10% to −10%; FVC worsened group, FVC decrease of <−10%. *CD20+ cells in the lymphoid follicle region: FVC improved group vs. FVC worsened group, p = 0.014. **CD20+ cells in the lymphoid follicle region: FVC stable group vs. FVC worsened group, p = 0.082.

### CD20+ Lymphocytes in Lung Tissue and CT Improvements

At 12 months after therapy, 17 patients exhibited >50% improvement in their chest HRCT scans (CT group 1), 19 patients exhibited <50% improvement (CT group 2), and 14 patients exhibited no improvement or worse findings (CT group 3). The follicular CD20+ lymphocyte counts showed an increasing trend from CT group 1 [129.67 (69.08–247.42)/0.1 mm^2^] to CT group 2 [231.00 (81.83–391.17)/0.1 mm^2^] to CT group 3 [312.00 (132.96–482.96)/0.1 mm^2^], but the difference was not statistically significant (p = 0.085) (Table 5). No significant differences were found in the other anatomic regions. There was no relationship between CD14+ lymphocytes and HRCT improvement.

**Table 5:** Relationship between CD20+ lymphocytes and CT improvements.

CD20+ Lymphocytes (counts/0.1 mm^2^)	CT improvements ^§^			*p* value
	CT-Group 1 (n = 17)	CT-Group 2 (n = 19)	CT-Group 3 (n = 14)	
Follicular region	129.67 (69.08–247.42)	231.00 (81.83–391.17)	312.00 (132.96–482.96)	0.085
Perivascular region	11.00 (6.58–22.33)	12.50 (5.50–18.50)	41.00 (4.71–47.24)	0.284
Interstitial region	7.17 (3.83–13.50)	7.17 (1.50–13.67)	9.25 (4.92–18.73)	0.527
Peribronchiolar region	6.67 (3.33–10.33)	7.50 (1.17–12.33)	6.50 (3.38–8.96)	0.982

^§^Treatment response according to CT findings after 1 year of steroid therapy. Group 1, CT exhibited >30% improvement in ground-glass opacities and reticular opacities; Group 2, CT exhibited <30% improvement; Group 3, CT exhibited no improvement or worsened findings.

### Survival of Patients with NSIP

**Figure 3:**
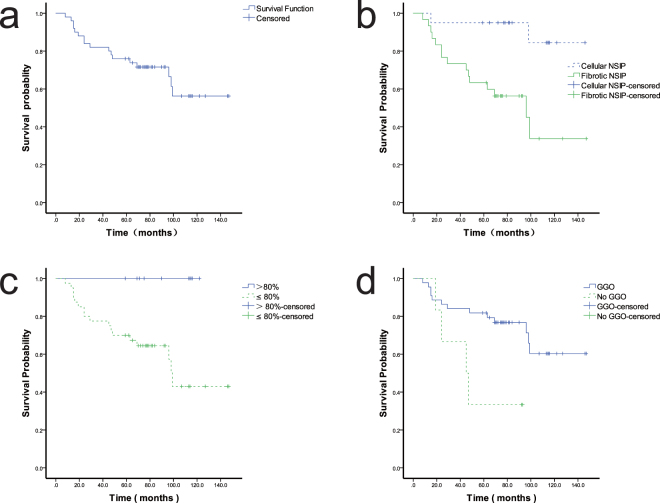
Kaplan–Meier survival curve for patients with nonspecific interstitial pneumonia (NSIP). (**a**) Overall survival of the patients. (**b**) Survival was better in patients with cellular than fibrosing NSIP (p = 0.002). (**c**) Survival was better in patients with a forced vital capacity of >80% than <80%. (**d**) Survival was better in patients with than without ground-glass opacity or consolidation on high-resolution computed tomography (p = 0.021).

The patients were followed up for a median of 75.5 months (range, 8–147 months). Seventeen patients died during the follow-up. Kaplan–Meier curves revealed better survival in patients with cellular NSIP than fibrosing NSIP (p = 0.002) (Fig. 3b) and better survival in patients with than without ground-glass opacity or consolidation on HRCT (p = 0.021) (Fig. 3d). Univariate analysis revealed a higher mortality risk in patients with a lower baseline FVC and higher erythrocyte sedimentation rate. Age, sex, clinical subtype of NSIP (CTD-NSIP, autoantibody-positive NSIP, and idiopathic NSIP), baseline DLCO, PaO_2_, and CD20+ lymphocyte infiltration in the lung were not correlated with survival. Multivariate analysis using Cox proportional hazard regression analysis revealed that the baseline erythrocyte sedimentation rate (hazard ratio, 1.030; 95% confidence interval, 1.007–1.053; p = 0.011) was an independent risk factor for survival and that the baseline FVC (hazard ratio, 0.947; 95% confidence interval, 0.908–0.988; p = 0.011) was a protective factor for survival.

## Discussion

In the present study, we found that CD20+ B lymphocytes were mainly distributed in the lymphoid follicles in lung specimens of patients with NSIP. Our study is the first to demonstrate that higher numbers of follicular CD20+ cells are present in fibrosing than cellular NSIP and that CD20+ cells within lymphoid follicles are negatively correlated with improvement in FVC after 1 year of therapy.

The NSIP pattern was histologically characterised by varying amounts of interstitial inflammation and fibrosis with a uniform appearance^1,2^. The lymphocytic infiltrate in NSIP exhibits elevated numbers of CD4/CD8 T cells with additional populations rich in CD20+ B cells^11^. Additionally, CD20+ B cells mainly accumulate in the lymphoid follicles in NSIP associated with polymyositis/dermatomyositis^29^, idiopathic NSIP^10^, and rheumatoid arthritis-associated NSIP^6,12^. We observed markedly increased numbers of B lymphocytes mainly distributed in the lymphoid follicles in NSIP, including CTD-NSIP, autoantibody-positive NSIP, and idiopathic NSIP, confirming previous reports of abnormal CD20+ B-cell aggregates within NSIP-affected lungs.

Although there is evidence of increased numbers of CD20+ B cells in ILD (including NSIP) and other chronic lung diseases, the precise mechanism is unclear. During an inflammatory process, leukocytes that have infiltrated the lung often assemble into structures known as inducible bronchus-associated lymphoid tissue (iBALT), which contains B-cell-predominant follicles surrounded by a parafollicular T-cell zone and dendritic cells^30,31^. iBALT is found in lung biopsies of patients with hypersensitivity pneumonitis, asthma, COPD, IPF and ILD associated with systemic sclerosis, rheumatoid arthritis, or Sjögren’s syndrome^4,7,8,30–34^. Overexpression of pro-inflammatory cytokines (interleukin-6 and its receptor, tumour necrosis factor, or interleukin-5) in the lungs has been shown to induce formation of iBALT in the absence of antigen or pathogen^4^. B-cell-activating factor of the tumour necrosis factor family (BAFF) contributes to the formation and expansion of pulmonary lymphoid follicles by promoting the survival and proliferation of follicular B cells^33^. The chemokines that drive the recruitment of B cells from the circulation include CCL21, and those involved in localisation within iBALT include CXCL12 and CXCL13 as well as CCL19 and CCL21^4,30,32^. Previous studies have shown that iBALT contributes to the local production of autoantibodies, correlates with local pathology, and participates in local pathogenesis in a wide variety of chronic lung diseases, although the precise mechanism remains poorly understood and deserves further investigation.

Although a few studies have shown the presence of highly abnormal intrapulmonary B-cell aggregates in patients with NSIP, as discussed above, no information has been available regarding the impact of the B-cell compartment on the clinical outcome of NSIP. Our study is the first to demonstrate that the follicular B cells in the lung are negatively correlated with improvement in FVC after therapy, indicating that a higher number of B cells is correlated with a lower response to therapy and a poorer clinical course. However, follicular B cells were not correlated with survival. Erythrocyte sedimentation rate was an independent risk factor for survival, indicating systemic inflamations might correlate with poor outcome, although the precise mechanism needs further investigation. Recent studies have indicated that B cells are also associated with disease progression in patients with IPF. The B lymphocytes in the lung are associated with a rapid decline of FVC^35^. Additionally, the plasma concentration of BLyS, an obligate factor for B-cell survival and differentiation, was shown to be elevated in patients with IPF and associated with poor survival^36^. There is a strong correlation between B cells and iBALT in the airway and the severity of airflow limitation in patients with COPD^32,33^. In addition, B lymphocytes play a role in autoimmune diseases. The percentage of activated peripheral blood B lymphocytes or the magnitude of this B-cell differentiation correlates with clinical activity in autoimmune diseases, such as rheumatoid arthritis and systemic lupus erythematosus^5,37–40^. Our results and those of previous studies appear to support the notion that B cells are correlated with poor outcomes and contribute to progression of various diseases such as ILD and autoimmune diseases.

Our study revealed that higher numbers of lymphoid follicular CD20+ B cells were associated with fibrosing NSIP, suggesting a possible contribution of the pathogenic potential of B cells to the fibrotic process. Although studies are limited and the precise pathophysiology remains poorly understood, evidence of a role of B lymphocytes in various fibrotic lung disease is growing^6,36^. Deficiency in the B-cell surface molecule CD19 in mice reduced the susceptibility to bleomycin-induced fibrosis, whereas CD19 overexpression in mice aggravated bleomycin-induced fibrosis, indicating a potential link between B cells and lung fibrosis^13^. A previous study of IPF revealed that lymphocytes (both T and B cells) were more numerous in advanced disease (with more fibrosis and honeycomb changes) than in early disease^9^. In a mouse model of silica-induced lung fibrosis, interleukin-10-producing regulatory B cells could control lung inflammation and exacerbate lung fibrosis by inhibiting the T-helper 1 response and modulating the T-helper balance^41^. B-cell proliferation and activation were significantly induced in irradiated lungs of fibrosis-prone mice, suggesting a possible role of B cells in radiation-induced fibrosing alveolitis^42,43^. B lymphocytes may contribute to the pathogenesis of fibrosing mediastinitis, and depletion of B cells is effective in treating this disease^44,45^. Together with previous findings, our study provides evidence that B cells play roles in fibrotic disease and that specific targeting of B lymphocytes may be explored as a potential treatment strategy for these fibrotic diseases.

Our study revealed that CD20+ B cells may play a role in the progression of fibrosing NSIP and is correlated with poorer therapeutic effects of in patients with NSIP. Thus, alternative therapies should be explored. Depletion of CD20+ cells may be a candidate therapy for NSIP. Rituximab is an effective depletor of CD20+ cells and is approved for the treatment of rheumatoid arthritis and systemic vasculitis^5^. Several studies have revealed that monoclonal anti-CD20 antibodies were effective in treatment of CTD-associated ILDs, particularly those that are resistant to steroid and cyclophosphamide treatments^46–49^. Thus, anti-B-cell agents (e.g., rituximab and others in development) may become therapeutic options for NSIP, which is characterised by inflammation, fibrosis, and autoimmune features^50^.

Our study has several limitations. First, it was retrospective in nature. Second, our sample size was limited and our results represent the experience of only a single centre. Therefore, a multicentre study with a larger cohort will be required to confirm our results. We will also need to clarify the relationship between B cells in the peripheral blood and B cells in the lung tissue.

## Conclusion

Greater infiltration of CD20+ cells within lymphoid follicles was correlated with the fibrosing pattern of NSIP and predicted less clinical improvement after treatment. The underlying mechanisms of this association are unknown, and further studies are required. These findings illuminate the potential for novel treatment regimens that specifically target B cells in patients with NSIP.
